# Latest Rational Treatment for Putrescent Gangrenous and Septic Root Canals

**Published:** 1907-04

**Authors:** C. B. Colson

**Affiliations:** Charleston, S. C.


### LATEST RATIONAL TREATMENT FOR PUTRESCENT GANGRENOUS AND SEPTIC ROOT CANALS.

By Dr. C. B. Colson, Charleston, S. C.

If there is a pathological or therapeutical subject or treatment in all therapeutical dentistry upon which we differ more individually in our methods and ideas, I cannot recall any other than the treatment of putrescent and septic root canals.

For many years the treatment of diseased pulps and the chambers and canals have been the most unsatisfactory part of our professional work. It has been a sad history and one in which there have been many failures. For the last thirty years we have made some progress in this line of treatment, some of us making a success with one method, but nearly every one using different methods and antiseptics.

Thirty years ago, on my advent into dentistry, carbolic acid and creosote were about the only treatment used. The contents of the canal, when possible, was drilled out and most likely it was impossible for either drill or barb to enter those canals, and there was no other treatment but this, for days and weeks, with carbolic acid until all odor seemed to pass away and the canal and chamber was then filled very generally in those days with carbolated cotton or with gutta percha.

It was quite often a failure and left many mouths with numerous fistulæ opening from devitalized teeth; a condition very rare today.

The difficulty was, that we could never be certain when a canal was truly clean and antiseptic; carbolic acid and creosote were not potent enough.

The barbs were then improved, and that helped us, and many blessed the Donaldson cleanser, and then to make this instrument of greater importance Dr. Calahan, of Cincinnati, gave us the great idea of using sulphuric acid to open the small canals and help cleanse them.

Still we did not have a potent antiseptic. The introduction of iodoform was a great advance and was used by many with success; it had its objectionable points, its odor and toxic effect and the liability in enlarged special openings to give the peculiar iodine taste and the objectionable odor to the breath, and again in very bad conditions with chronic suppuration it failed and was not as potent as carbolic acid; it was a better preventative to sepsis than a destroyer.

Then came the two schools, one for using the non-coagulents which caused a long discussion not necessary to explain here. Many ideas were brought out that gave excellent results, in some hands they were particularly successful in almost every case, while with others they were dismal failures. The Evan's Root Dryer; the Blair Atomizer with idoform vapor and last but not least Kalium Natrium, and sodium and potasa, and other ideas and drugs too numerous to mention.

Today I feel safe in saying that over fifty per cent. of the profession are using carbolic acid with fair success with the assistance of better instruments, acids, hot air and antiseptic filling material.

We had felt that our treatment was empirical; after the germ theory was more fully understood many of us felt that the treatment was rational and every idea was to destroy the septic germ.

But little do we know today, of the true phenomena of the decomposition of pulp tissue or its various stages of disintegration. Our treatment with the most potent of germicides and antiseptics must still be empirical until we know more of both the living and dead pulps.

True, the chemist positively tells us that the living pulp contains the following six elements: Carbon, hydrogen, oxygen, nitrogen, sulphur and iron; but with all that nice analysis no one yet has authoritatively stated there is fat in the tissues of the pulp.

The dental chemist is far behind in his work; the bacteriologist has outstripped him in giving us the greatest help.

The object of this paper is not to tell you of any great idea of mine but to help spread the last useful idea that I consider has the greatest value of any in therapeutical dentistry of this or the last century.

Septic or putrescent root canals with chronic suppurating fistulas or abscesses have no more worry for me, or any of us who know of the message I am about to deliver. I am only passing it on.

"Is there a blessing known
Pass it on,
'Twas not for you alone
Pass it on.
Let it travel down the years,
Let it dry another's tears,
'Till in heaven the deed appears,
Pass it on."

The chemistry of pulp decomposition, its fermentations, gaseous generations, its sequelæ, its six elements, its nitrogenous or non-nitrogenous substances, its carbo-hydrates and fats, whether it coagulates in the tubuli or not, or what are its end products no more need worry you, there is a rational potent germicidal and antiseptic treatment potent and powerful enough to almost instantly correct and make all such cases pure and painless.

This agent is Formaldehyde. Formaldehyde has been known as a potent germicide for many years and lately it has been in general use in the schools of preventive medicine and hygiene as the most potent of all germicides and the most adaptable, being gaseous in form.

Ten years ago, when it came into general use, I endeavored to use it in dentistry in various forms and percentages, but it was so unstable, my knowledge of its power so little that I caused several patients many moments of acute suffering and I became discouraged and discontinued its use.

At the International meeting at St. Louis, I heard a paper read by Dr. Buckley, of Chicago, that set me thinking again of the value of formaldehyde. The paper came under Section III, Chemistry an Metallurgy, published in Transactions, Vol. II, page 10. It was the most scientific and valuable of all the papers read at the International meeting.

In all cases where we find a tooth who's pulp is undergoing decomposition, dead, mummified, gangrenous, or with fistulous openings or abscessed, open up the chamber and seal within, with good cement, without pressure, a preparation of formaldehyde and the results will be such as you never have had before.

Formaldehyde, I will say to those who are not familiar with it, is a gaseous agent; its common commercial form is a forty per cent. aqueous solution known in the pharmacopœia as formalin.

The early experimenters used the preparation in one, two and five per cent. solution with water. In this form it was not stable or a success. We have since found it to be potent with certain forms of carbolic acid, but it does not combine well with pure carbolic acid.

It was used very successfully with Tricresol equal parts. Further experiment found that the best results yet were from the following preparation of this germicide and if used with judgment was perfectly safe and its effect wonderful.

Rx. Take two drachms of formalin and creosote each, add together; this will form a dark mixture. Slowly add pure alcohol and shake well and keep adding the alcohol until the the whole becomes clear.

In all cases the mode of using it is almost the same. Use the instrument as little as possible, simply to open the chamber well; if it can be done without danger of forcing out the contents of the canal through the apex; if there is danger of this, just get a small opening to the chamber and saturate a piece of cotton with the above preparation, place same over the opening to the chamber and seal the cavity with a cement without pressure. If you can get the chamber fully open one treatment is all the tooth seems to need, but I often, as a precaution after removing the cement on the return of the patient, remove the contents of the canals and and renew the treatment. My successes have been all that I could wish.

For instance, in the dead teeth that come to us for treatment, that are elongated from acute pericementitis and nearing the stage of abscess, this treatment invariably aborts the abscess if the suppurative stage has not been reached.

Another class of teeth upon which it acts like a charm are the chronically sore teeth that remain sore after thorough scouring and the most extraordinary course of treatment; too sore for comfort or use. One treatment after the canals are opened is all that is needed.

Again, there is a class of teeth that we meet in elderly persons, worn off almost to the cervex and almost the entire canal filled in by a retiring pulp. These teeth give us much trouble to treat; they get sore and it often takes months to get them in a normal state, but I have never had a failure in nearly two years and have had a large number to crown and bridge.

Now, a word of caution as to its dangers and disadvantages.

Formalin is one of the most irritating drugs we have to the human economy. When it touches either skin or mucous membrane it causes acute pain difficult to relieve, and therefore it must be used with caution.

If forced through the apical foramen it causes very acute pain and soreness of the tooth for several hours, and if in a great quality, will cause an abcess.

A canal may be swabbed out with a small quantity of cotton wrapped on a broach, but it must not be pumped, as this is very unnecessary anyhow.

It is not the liquid preparation that does the work, it is the potent gas that escapes and destroys all germs in the canals and also permeating the tubuli and destroying every spore or germ. As a deodorizer there is nothing to equal it, the most putrescent and noxious smelling pulp chamber in one treatment becomes perfectly pure.

There has been very little yet written on formaldehyde in our literature for the past year, but I have noticed my mail flooded with numerous nostrums in small forms, pastes, tablets and putties for performing the very same offices I have here brought to your attention; all of them claim to have a small quality of formaldehyde, but it is very cautiously mentioned; they seem modest to give the true credit to the agent that does the work. I have tried them all, both from samples and supplies purchased for experimental purposes. My brothers, as soon as a good idea is brought out in dentistry there are always awaiting a lot of harpies to feed on it. They are ready to sell to you at some enormous price something that they have stolen.

They are ever ready to grasp an idea to make the mighty dollar. They "pass it on" for the dollar alone.

I want a few words more to condemn this class of men and also the dentist who continually patronize the proprietary article, rather than make their own office preparations.

You know little of what you are using in the ready made preparations. You are most damnably empirical and have no chance or opportunity to improve or add your own ideas and make changes according to your experiences.

I most heartily condemn these preparations, as I have before in your meeting, the ready-made cocaine preporations that rob you and which you can put up yourself for fifteen cents, and you are deceived at the same time as to the quality or percentage of the various drugs used, and if they get you into trouble, you deserve to stay there, and no self-respecting dentist should give aid to a quack, who persists in being a quack.

My brothers, formaldehyde is a blessing to dentistry. I know that some of you use it and know of its advantages, and its particular potency. "Let us pass it on."

I hope to have a good discussion, and have endeavored not to exhaust the subject.

